# Congenital Bilateral Cataracts Associated with CRYBA1: A Familial Case Report Highlighting Genetic Diagnosis, Surgical Management, and Lifelong Glaucoma Surveillance

**DOI:** 10.3390/ijms27219978

**Published:** 2026-11-07

**Authors:** Mariya R. Ahmed, Tehmeena Akhter, Firoz Kabir, Christina Kilby, Andrew Nguyen, Osamah J. Saeedi, Janet Leath Alexander

**Affiliations:** 1 School of Medicine, University of Maryland, Baltimore, MD 21201, USA; mahmed@som.umaryland.edu (M.R.A.);; 2 Department of Otorhinolaryngology-Head & Neck Surgery, School of Medicine, University of Maryland, Baltimore, MD 21201, USA; takhter@som.umaryland.edu (T.A.);; 3 Centre of Excellence in Molecular Biology, University of the Punjab, Lahore 53700, Pakistan; 4 Department of Biochemistry and Biotechnology, University of Science and Technology Chittagong, Chittagong 4202, Bangladesh; 5 Department of Ophthalmology and Visual Sciences, School of Medicine, University of Maryland, Baltimore, MD 21201, USA

**Keywords:** congenital cataract, *CRYBA1*, p.(Gly91del), aphakia, pediatric cataract surgery, glaucoma, genetic testing

## Abstract

Congenital cataracts are an important cause of childhood visual impairment and require timely treatment to preserve visual development. Their management includes surgical removal of visually significant lens opacities, prompt optical rehabilitation, and lifelong surveillance for complications such as glaucoma and strabismus. We report a male infant with dense bilateral congenital cataracts and a multigenerational maternal history of congenital cataracts. Exome sequencing identified a heterozygous *CRYBA1* variant, NM_005208.5:c.272_274del, p.(Gly91del), which was confirmed by Sanger sequencing in the proband and his affected mother. Gly91 is highly conserved, and structural modeling places the residue within a beta/gamma-crystallin Greek-key domain, where its deletion is predicted to disturb local protein folding and stability. The infant underwent sequential bilateral cataract extraction one week apart, and his aphakia was managed with contact lenses followed by spectacles. Infrequent intermittent esotropia was observed during follow-up, but no elevation of intraocular pressure or evidence of optic nerve damage was detected. At 3 years 7 months of age, best-corrected visual acuity with aphakic spectacles was 20/50 in each eye and 20/20 binocularly. The mother, who had undergone cataract surgery during infancy, subsequently developed advanced glaucoma of both eyes with marked retinal nerve fiber layer thinning. This case demonstrates the value of integrating molecular diagnosis with timely surgical treatment, optical rehabilitation, and long-term postoperative surveillance. Identification of the familial *CRYBA1* variant confirmed the molecular diagnosis and supports genetic counseling, while the mother’s clinical course underscores the need for lifelong glaucoma monitoring following congenital cataract surgery.

## 1. Introduction

Congenital cataracts are lens opacities present at birth or developing during early infancy, and they represent an important, preventable cause of childhood visual impairment worldwide [1,2]. Visually significant cataracts interfere with the formation of a clear retinal image during the critical period of visual development, placing affected children at substantial risk of deprivation amblyopia and resultant permanent visual loss if treatment is delayed [1,3]. The burden is particularly high in settings where access to neonatal screening, pediatric ophthalmology services, and timely surgical care are limited [1,2].

The etiology of congenital cataracts is clinically and genetically heterogeneous. Cataracts may occur as isolated ocular abnormalities or as part of chromosomal, metabolic, infectious, or multisystem disorders [4,5]. A substantial proportion of bilateral congenital cataracts have a genetic basis, with autosomal dominant inheritance frequently observed in isolated familial cases [4,5]. Disease-associated variants have been identified in genes encoding crystallins, connexins, membrane proteins, transcription factors, and components of the lens cytoskeleton [4,5]. Crystallins constitute the major structural proteins of the lens and are essential for maintaining its transparency, refractive properties, and long-term protein stability [6]. *CRYBA1* encodes the closely related βA3- and βA1-crystallins through alternative translation initiation. Pathogenic *CRYBA1* variants can impair crystallin folding, stability, or solubility, thereby promoting protein aggregation and lens opacification [6,7,8].

The management of visually significant congenital cataracts requires more than removal of the opaque lens. While early surgery reduces the risk of irreversible amblyopia, intervention during infancy carries long-term challenges including aphakia, refractive changes with ocular growth, visual-axis opacification, strabismus, and secondary glaucoma [2,3,9,10]. Decisions regarding the timing of surgery, primary intraocular lens implantation, and postoperative optical correction must therefore be individualized to age, cataract morphology, visual potential, and clinical circumstances. Following surgery, prompt optical rehabilitation and amblyopia therapy are essential, and lifelong surveillance is required because complications, particularly glaucoma, may develop many years after an initially successful procedure [3,9,10].

Genetic testing is increasingly valuable in evaluating children with bilateral congenital cataracts. Establishing a molecular diagnosis can clarify disease etiology, distinguish isolated from syndromic forms, inform offspring recurrence-risk assessment, guide testing of relatives, and support reproductive counseling [4,5]. In this report, we describe a male infant with dense bilateral congenital cataracts and a multigenerational maternal history of cataract who was found to carry the recurrent heterozygous *CRYBA1* variant NM_005208.5:c.272_274del, p.(Gly91del). The child underwent sequential bilateral cataract extraction and postoperative optical rehabilitation. The advanced glaucoma experienced by his affected mother following her own cataract surgery in infancy further emphasizes the importance of integrating molecular diagnosis, surgical treatment, and lifelong postoperative surveillance in the management of hereditary congenital cataracts.

## 2. Case Presentation

### 2.1. Clinical Presentation and Family History

**Figure 1:**
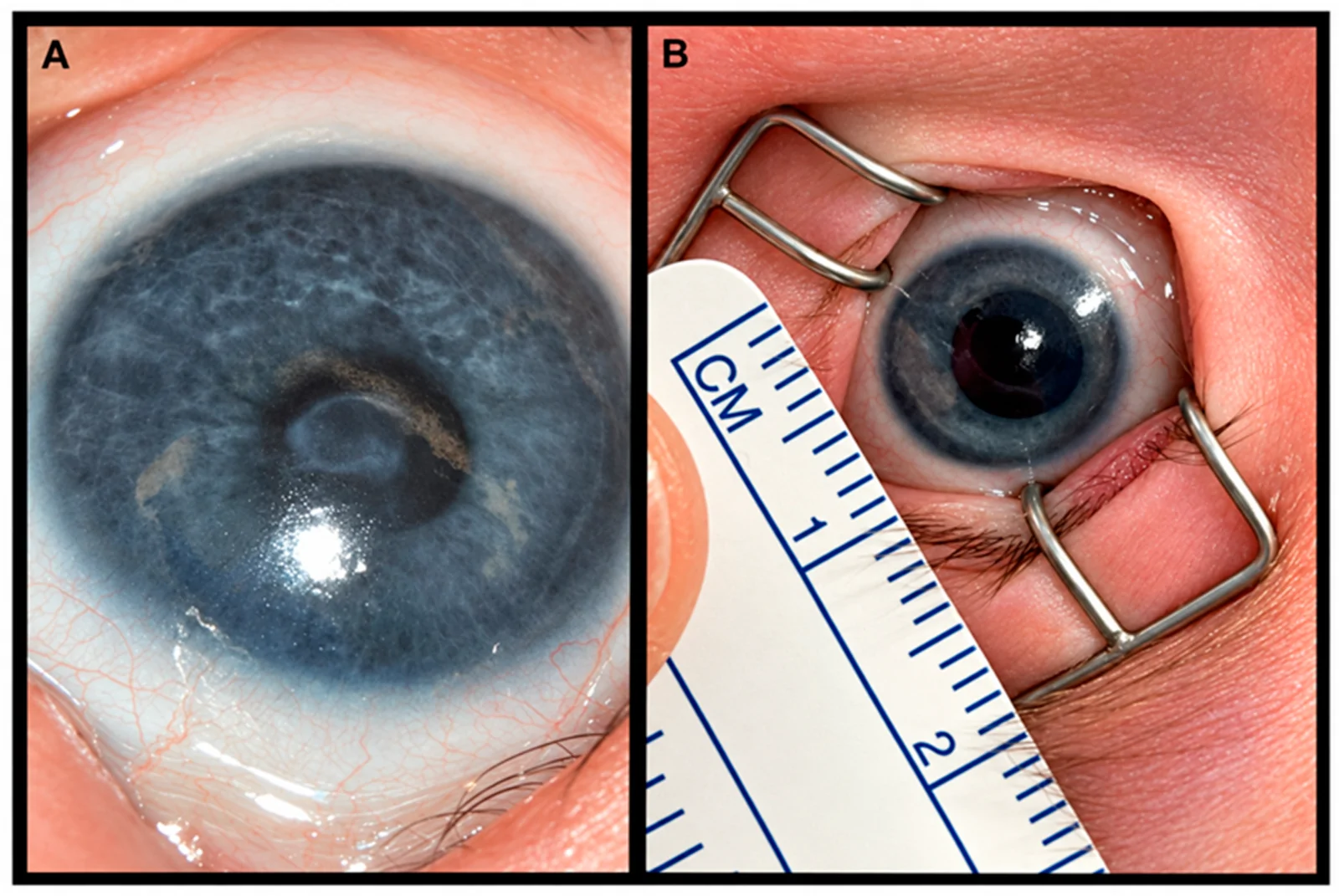
Preoperative and Postoperative photographs of the proband’s right eye. (**A**) Preoperative image demonstrating a dense congenital cataract with central opacification. (**B**) Postoperative image showing a clear cornea and an aphakic pupil following cataract extraction. A ruler is included for scale.

A male infant (proband, Eye-83) was noted to have an absent red reflex in both eyes on newborn examination. He was born at 37 weeks’ gestation (birth weight 2.78 kg) after an uncomplicated delivery. Dense bilateral congenital cataracts were confirmed by an outside ophthalmologist, and he was evaluated at our institution at 5 weeks of age. The proband and his mother are White and non-Hispanic, as reported by the family. Apart from the ocular findings, he has been healthy: routine pediatric examinations have shown no systemic findings suggestive of a syndromic, metabolic, or infectious cause, and his growth and development have been normal. The lens opacities were considered visually significant and posed a substantial risk of deprivation amblyopia. Preoperative examination of the right eye demonstrated a dense central lens opacity, as shown in Figure 1A. A persistent pupillary membrane was present in the right eye, with miosis; topical atropine 1% once daily was started preoperatively to help release the iris–lens adhesions. There was no iris or chorioretinal coloboma and no other sign of persistent fetal vasculature, such as a persistent hyaloid vascular stalk, a retrolental fibrovascular membrane, or elongated ciliary processes. Axial lengths were 17.20 ± 0.02 mm in the right eye and 17.21 ± 0.01 mm in the left eye, within the normal range for age and arguing against microphthalmia. Ultrasound biomicroscopy (UBM) showed open iridocorneal angles and a normal ciliary body configuration, without a retrolental membrane (Figure S1). The right iris had adhesions (the left did not), and bilaterally the lens had dense posterior opacity, but the posterior capsule did not have a stalk or any obvious defects.

### 2.2. Surgical Management and Clinical Follow-Up

Because of the severity of the bilateral cataracts and the risk of irreversible amblyopia, sequential cataract surgery was performed at 8 weeks of age in the left eye and at 9 weeks of age in the right eye. Sequential rather than same-day bilateral surgery was chosen to avoid the small but potentially devastating risk of bilateral postoperative infection, and the left eye was operated on first because of the miosis in the right eye. Each procedure was performed under general anesthesia and included an examination under anesthesia. Through two 1.2 mm paracentesis incisions, the anterior capsule was stained with trypan blue, a 4.5 mm continuous curvilinear anterior capsulorhexis was created, and the lens was removed by irrigation and aspiration. A primary posterior capsulotomy was then created and enlarged with a 23-gauge vitrector, followed by anterior vitrectomy. In the right eye, the pupillary membrane was excised with vitreoretinal scissors after viscodissection failed to release it, and five iris hooks were placed to enlarge the pupil. No intraocular lens was implanted; the family had been counseled preoperatively that both eyes would be left aphakic, with secondary IOL implantation to be considered after further ocular growth. There were no intraoperative complications. A +32.0 D silicone elastomer aphakic contact lens was placed on each eye at the end of surgery. Postoperative examination demonstrated a clear cornea and pupil without evidence of residual central lens opacity or other immediate surgical complications (Figure 1B). Postoperative dilated fundus examination showed healthy-appearing optic nerves with a cup-to-disc ratio of 0.1 in each eye, normal retinal vasculature and maculae, and no chorioretinal coloboma.

**Figure 2:**
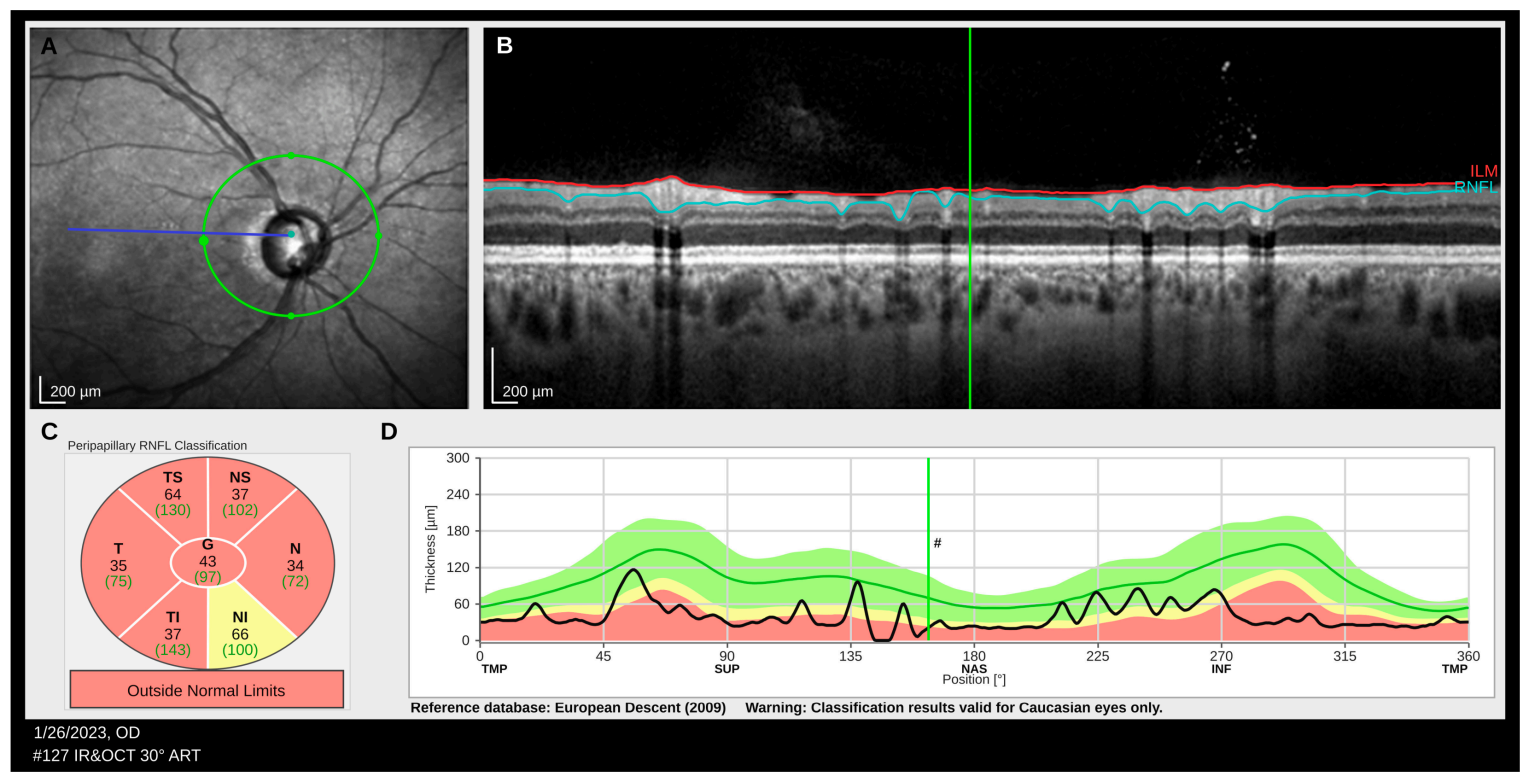
OCT analysis of the proband’s mother with advanced glaucoma. Spectralis OCT (Heidelberg Engineering) peripapillary RNFL scan of the right eye, obtained at 40 years of age; OCT could not be obtained in the left eye. (**A**) En face infrared image showing the circular scan pattern centered on the optic nerve head for peripapillary RNFL measurement. (**B**) Cross-sectional OCT B-scan along the peripapillary circle with automated segmentation of the internal limiting membrane (red line) and the posterior boundary of the RNFL (blue line). (**C**) Sectoral RNFL thickness classification compared with the instrument’s European Descent (2009) reference database; each sector shows the measured thickness (µm), with the reference mean in parentheses. The global RNFL thickness is reduced to 43 µm (reference: 97 µm). The temporal superior (TS), nasal superior (NS), temporal (T), nasal (N), and temporal inferior (TI) sectors are outside normal limits (red), and the nasal inferior (NI) sector is borderline (yellow). (**D**) RNFL thickness profile (black line) plotted against normative percentiles (green = within normal limits, yellow = borderline, red = outside normal limits). The profile demonstrates diffuse thinning, particularly in the superior and inferior quadrants, with extensive deviation into the <1st percentile range. #: Automatically generated symbol embedded in the software output.

The proband’s mother, now 43 years of age, underwent bilateral cataract surgery at 1 to 2 months of age and has remained aphakic, with spectacle correction. Glaucoma was diagnosed shortly after this surgery, during infancy, and she has required treatment for approximately four decades. She underwent glaucoma surgery during elementary school (at approximately 7 to 10 years of age) and again during adolescence. In the left eye, she subsequently underwent two glaucoma drainage implant procedures and a penetrating keratoplasty; in the right eye, a glaucoma drainage implant was placed in her late 30s. Her current medical therapy consists of topical bimatoprost, dorzolamide-timolol, and brimonidine in both eyes, together with topical prednisolone acetate. The only available Intraocular Pressure (IOP) measurement, obtained on treatment at 40 years of age, was 20 mmHg in each eye. Her glaucoma is advanced in the right eye and end-stage in the left eye. At 43 years of age, best-corrected visual acuity in the right eye ranged from 20/125 to 20/320 depending on the chart and contrast polarity, and the left eye had no light perception, having declined from bare light perception at 40 years of age. Goldmann perimetry of the right eye showed generalized constriction with near-total loss of the superior field; contrast sensitivity was severely reduced (Pelli-Robson, 0.85 log units binocularly), and she is legally blind by visual acuity. Optical coherence tomography (OCT) of the right eye, obtained at 40 years of age, demonstrated marked diffuse retinal nerve fiber layer (RNFL) thinning, with a global thickness of 43 µm compared with a normative value of 97 µm. Severe thinning was present in multiple peripapillary sectors, consistent with advanced glaucomatous optic neuropathy (Figure 2 and Table 1). Peripapillary OCT could not be obtained in the left eye.

As planned preoperatively, the contact lenses were replaced at 3 months of age, approximately one month after the second surgery, with full-time single-vision aphakic spectacles. Spectacles were prescribed based on the full cycloplegic refraction with slight additional plus power to favor near vision, because bifocals are not typically used in infants; a bifocal add is planned for later childhood to compensate for the loss of accommodation. Regular ophthalmologic follow-up, initially at 3-month intervals and subsequently at 6-month intervals, included assessment of visual behavior, refractive correction, ocular alignment, intraocular pressure, and optic nerve appearance. The postoperative course was uncomplicated: the pupillary membrane resolved, the visual axis remained clear, IOP was normal at every visit from one month after surgery onward, and no additional surgery was required.

At the most recent examination, at 3 years 7 months of age and approximately 3.5 years after surgery, best-corrected visual acuity with aphakic spectacles, measured with a linear Snellen chart, was 20/50 in each eye and 20/20 binocularly; monocular testing was limited by the child’s initial reluctance to accept occlusion. IOP measured by rebound (iCare) tonometry was 13 mmHg in the right eye and 14 mmHg in the left eye; the corneas and anterior vitreous were clear, and the optic discs were normal, with a cup-to-disc ratio of 0.1 in each eye. Intermittent esotropia had been noted during follow-up but was infrequent, and its apparent size was exaggerated by the magnification of the high-plus spectacles. On examination, he was orthotropic at distance and near by alternate cover testing and by Krimsky testing, with no fixation preference and no nystagmus. Amblyopia therapy has not been required. Continued monitoring was recommended, with occlusion therapy and strabismus treatment to be considered if ocular misalignment or asymmetric visual development became clinically significant.

A comparison of the genetic findings, clinical manifestations, and postoperative outcomes of the proband and his affected mother is presented in Table 2. Although both individuals carry the same *CRYBA1* variant and presented with bilateral congenital cataracts, their postoperative clinical courses differed, with advanced glaucoma observed in the mother but not detected in the proband during the available follow-up period.

### 2.3. Molecular Diagnosis and Genetic Findings

**Figure 3:**
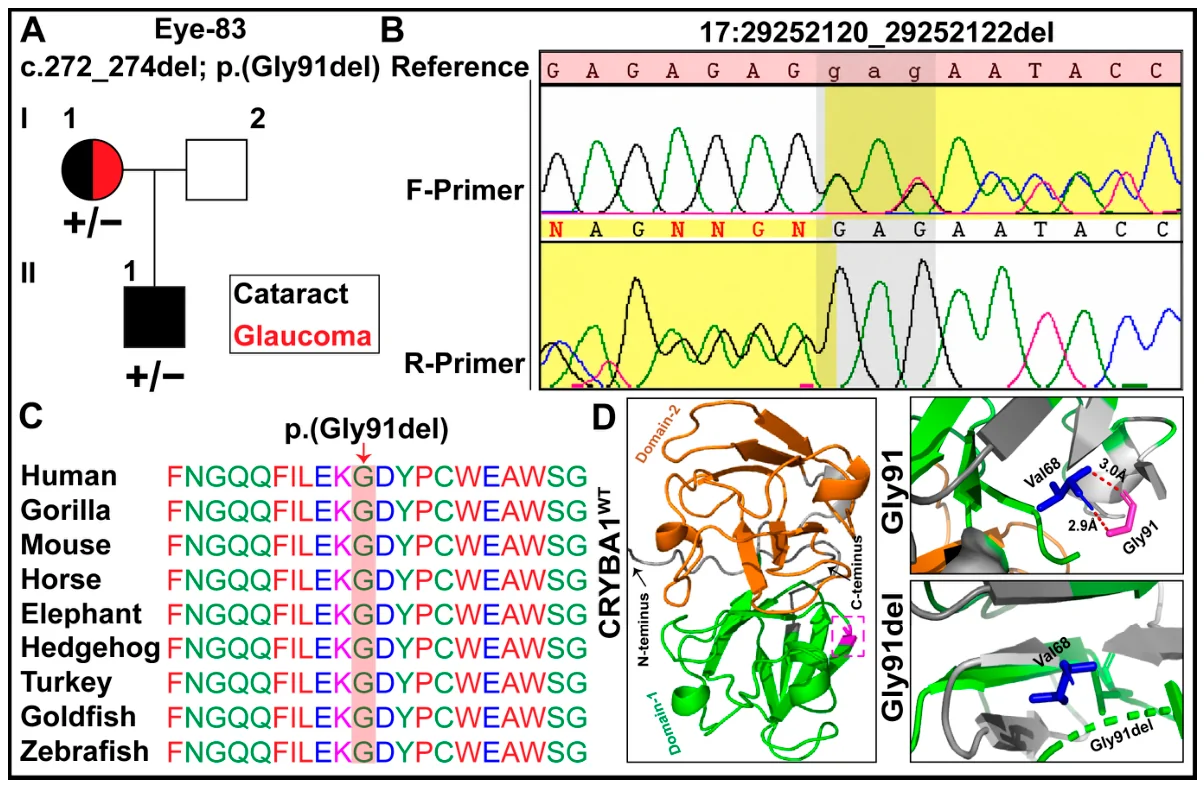
Identified variant of CRYBA1 predicted to be damaging, deleting a conserved glycine residue and impacting the protein secondary structure. (**A**) Pedigree of family Eye-83 showing the available family relationships and the inheritance of congenital cataracts. Black shading indicates congenital cataracts, whereas red shading indicates documented glaucoma. The proband presented with bilateral congenital cataracts without evidence of glaucoma, while his affected mother developed advanced glaucoma following cataract surgery during infancy. The heterozygous *CRYBA1* p.(Gly91del) variant was confirmed in the proband and his mother. (**B**) Sanger sequencing shows a 3 bp deletion in *CRYBA1* in a heterozygous pattern. (**C**) Clustal Omega alignment revealed that Gly91 is a highly evolutionarily conserved residue, suggesting deletion of this amino acid is damaging to the protein. (**D**) Three-dimensional structural representation of CRYBA1 obtained from AlphaFold and visualized using PyMOL v3.1. The position of Gly91 within the second β/γ-crystallin Greek-key motif is highlighted. The enlarged view illustrates the local structural environment surrounding Gly91 and its spatial relationships with neighboring amino acid residues. Dashed lines indicate putative hydrogen-bond interactions, with measured interatomic distances expressed in Å. Domain 1: Beta/gamma crystallin ‘Greek key’ 1, 2; Domain 2: Beta/gamma crystallin ‘Greek key’ 3, 4. N-terminus: Amino terminus; C-terminus: Carboxy terminus.

Exome sequencing identified a heterozygous three-base-pair in-frame deletion in *CRYBA1*, NM_005208.5:c.272_274del, predicted to result in deletion of glycine at amino acid position 91, NP_005199.2:p.(Gly91del). The variant was confirmed by bidirectional Sanger sequencing in the proband and his affected mother, supporting maternal inheritance and cosegregation with congenital cataracts in the two genetically evaluated affected individuals. The available pedigree and sequencing results were consistent with autosomal dominant inheritance (Figure 3A,B).

The proband had a notable maternal family history of congenital cataracts spanning multiple generations. According to the mother’s reported family history, congenital cataracts affected her father, four siblings, a niece, and a nephew. This familial distribution was consistent with an autosomal dominant pattern of inheritance. However, detailed clinical information, precise pedigree relationships for some extended family members, and molecular testing were unavailable for these relatives. Therefore, the pedigree (Figure 3A) represents family members for whom sufficient relationship information was available, while the additional reported affected relatives are described in the clinical history. The CRYBA1 p.(Gly91del) variant was genetically confirmed only in the proband and his affected mother among the affected individuals tested.

**Figure 4:**
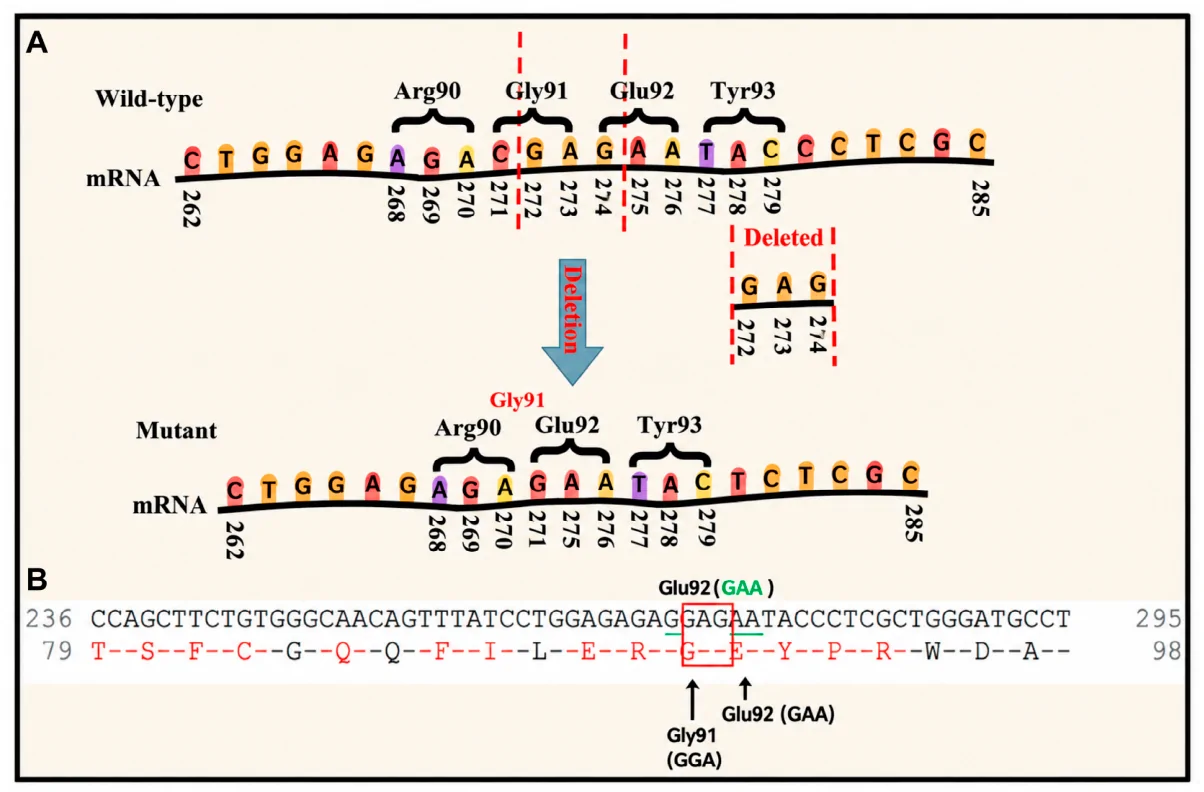
In-frame deletion of Glycine 91 (G91) in CRYBA1. (**A**) Glycine 91 (G91), situated at the edge of a β-sheet in the crystallin protein, is essential for maintaining the structural integrity and proper folding of the β-sheet. Its deletion disrupts the protein’s secondary structure and stability. (**B**) In the wild-type sequence, codons at positions 268_279 encode Arg90 (AGA), Gly91 (GGA), Glu92 (GAA), and Tyr93 (TAC). However, in the mutant mRNA, an in-frame deletion of the triplet GAG (c.272_274del) removes the last two nucleotides of the GGA codon (Gly91) and the first nucleotide of the GAA codon (Glu92). This eliminates the Gly91 residue without altering the downstream reading frame, resulting in Glu92 (GAA) directly following Arg90. The absence of Gly91 impairs protein folding and function.

The p.(Gly91del) variant results in the deletion of a single amino acid without altering the downstream reading frame (Figure 4). Gly91 is highly conserved across vertebrate species and is located within the second β/γ-crystallin Greek-key motif of CRYBA1 (Figure 3C,D). Structural analysis using an AlphaFold-predicted protein model visualized in PyMOL identified the position of Gly91 within the β-sheet architecture and its spatial relationships with neighboring residues. The deletion of this conserved residue may perturb local structural organization and compromise crystallin folding and stability, consistent with previously published structural and functional investigations of the same variant [8,11]. Consistent with this interpretation, our structural visualization highlights the position of Gly91 and its local interaction environment (Figure 3D). However, the present computational analysis does not independently establish changes in global protein conformation, thermodynamic stability, or aggregation.

The identified *CRYBA1* variant, NM_005208.5, c.272_274del, p.(Gly91del), has previously been classified as pathogenic in ClinVar (Variation ID: 425127). This recurrent in-frame deletion has been reported in multiple unrelated families affected by autosomal dominant congenital cataracts. Previous functional studies have demonstrated that the deletion adversely affects crystallin folding, solubility, and protein stability, supporting its pathogenic role. In the present family, the variant was detected in the affected proband and his affected mother and was absent from the unaffected father, consistent with maternal transmission. The published genetic and functional evidence, together with the findings from our family, supports the established association of p.(Gly91del) with congenital cataracts.

The variant was absent from the population database examined and had high evolutionary conservation and computational pathogenicity scores, including a CADD PHRED [12] score of 40, a GERP++ score of 5.88, a phyloP 100-way vertebrate score of 9.54, and a phyloP 470-way mammalian score of 8.669. Table 3 summarizes the complete variant-interpretation evidence.

## 3. Discussion

This case highlights the importance of integrating genetic diagnosis, early surgical intervention, optical rehabilitation, and long-term ophthalmologic surveillance in the management of hereditary bilateral congenital cataracts [2,4]. The proband’s cataracts were detected by an absent red reflex at birth, and he underwent sequential bilateral cataract extraction, followed by optical correction with contact lenses and subsequently spectacles. Molecular testing identified a recurrent heterozygous *CRYBA1* variant (NM_005208.5:c.272_274del, p.(Gly91del)) that was also present in his affected mother, consistent with the multigenerational autosomal dominant inheritance pattern observed in the family. Notably, the mother’s history of advanced glaucoma following cataract surgery during infancy underscores the potential long-term consequences of postoperative complications and the need for continued ophthalmologic surveillance.

Bilateral congenital cataracts are more likely than unilateral cataracts to have an identifiable hereditary, syndromic, or systemic cause [14], and the extent of the diagnostic workup should be guided by the family history and a general pediatric examination. When there is no family history, or when the child has dysmorphic features, developmental delay, or other systemic findings, evaluation commonly includes serologic testing for congenital infections (toxoplasmosis, rubella, cytomegalovirus, herpes simplex virus, and syphilis), screening for galactosemia with urine reducing substances and red blood cell enzyme assays, serum calcium, phosphate, and glucose, urine amino acids when Lowe syndrome is suspected, and genetics consultation, with neuroimaging when central nervous system involvement is suspected [5,14]. In contrast, an otherwise healthy child with a clear autosomal dominant history of isolated cataract, as in this family, is unlikely to benefit from broad infectious or metabolic testing [14]. Accordingly, extensive infectious, metabolic, or neurologic investigations were not performed in the proband. Targeted next-generation sequencing panels or exome sequencing are increasingly used early in this evaluation, because a molecular diagnosis can confirm an isolated hereditary etiology, reveal unsuspected syndromic or metabolic disease, and reduce the need for nontargeted investigations [10,15].

Early removal of visually significant congenital cataracts is essential to prevent prolonged visual deprivation and irreversible amblyopia. However, surgical timing requires careful consideration, as delayed intervention may compromise visual development, whereas surgery during early infancy is associated with an increased risk of postoperative complications, particularly glaucoma [16]. In the present case, lens aspiration with primary posterior capsulotomy and anterior vitrectomy, followed by aphakic optical correction, was consistent with established management of infantile cataract. In very young children, posterior capsule opacification develops rapidly and almost inevitably when the posterior capsule is left intact, so primary posterior capsulotomy with anterior vitrectomy is considered a routine step in infant cataract surgery [17]. Primary IOL implantation was deferred because current evidence does not show a visual benefit that outweighs its risks in infants. In the Infant Aphakia Treatment Study, which randomized infants aged 1 to 6 months undergoing unilateral cataract surgery, visual acuity did not differ between contact lens and primary IOL correction at 1 year or 4.5 years of age, whereas more children in the IOL group had adverse events (81% vs. 56%) and additional intraocular operations (72% vs. 21%) by 5 years of age [18,19]. In children aged 2 years or younger, including those with bilateral cataracts, the population-based IoLunder2 cohort found that primary IOL implantation did not improve 5-year visual outcomes or protect against glaucoma and increased the risk of early reoperation [20]. In addition, rapid and variable axial growth during infancy makes the long-term refractive outcome of a primary IOL difficult to predict [2,5]. In bilateral aphakia, aphakic spectacles are a practical alternative to contact lenses, because the similar correction in both eyes avoids the aniseikonia that limits spectacle use in unilateral aphakia. Secondary IOL implantation may be considered at a later age depending on visual development, refractive stability, and clinical assessment [2].

**Figure 5:**
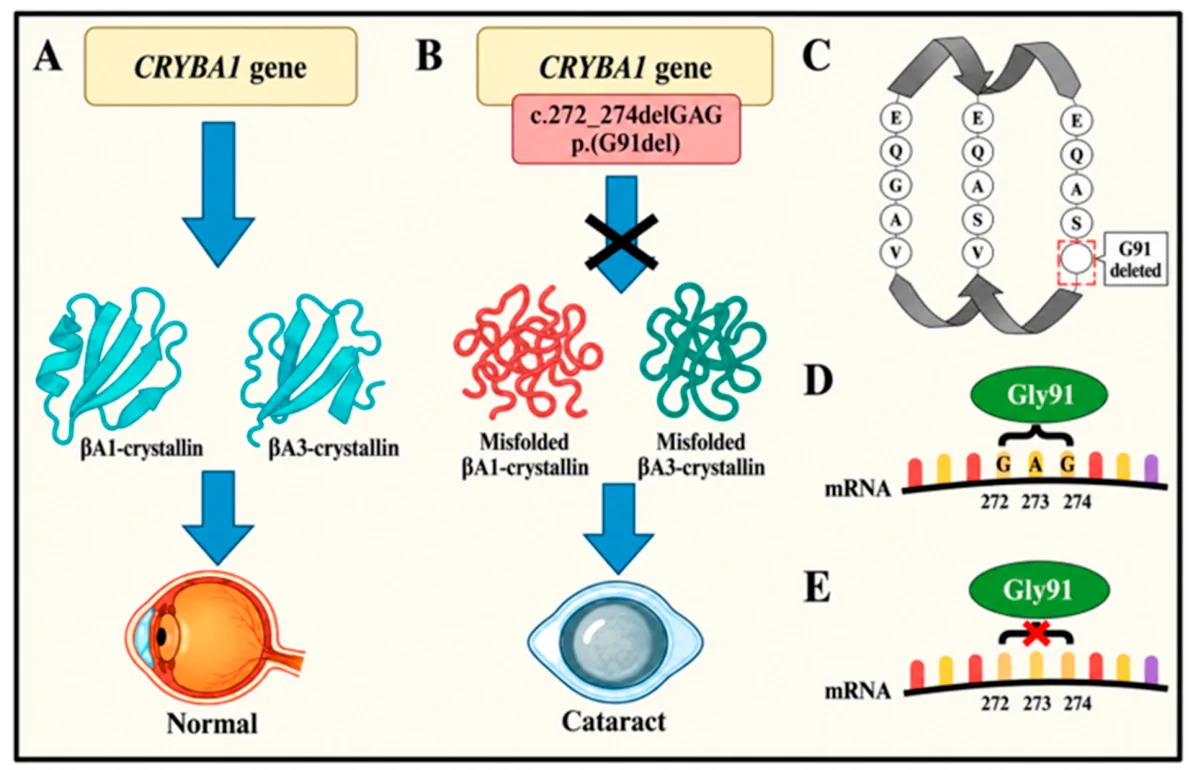
Proposed molecular mechanism of cataract formation associated with the *CRYBA1* p.(Gly91del) variant. (**A**) The Wild-type *CRYBA1* gene encodes βA1- and βA3-crystallins, which contribute to lens transparency and maintenance of its refractive properties through proper protein folding and organization. (**B**) The in-frame deletion c.272_274del, p.(Gly91del), is predicted to disrupt the structural integrity of βA1- and βA3-crystallins, potentially affecting their stability and solubility and promoting protein aggregation and lens opacification. (**C**–**E**) Schematic representations of the Gly91 deletion and the corresponding wild-type and mutant sequence configurations. The figure illustrates a literature-supported molecular mechanism rather than experimentally measured protein aggregation in the present study.

The identified *CRYBA1* variant has previously been associated with autosomal dominant congenital cataracts in families from diverse populations. Khidiyatova et al. [6] reported the same variant in Russian families with congenital cataracts, with its pathogenic classification supported by genetic analyses, including Sanger sequencing. The variant has also been documented in Chinese, European, and Iranian families, indicating its recurrence across different populations [6,21]. Despite a shared molecular etiology, affected individuals exhibit phenotypic variability, with nuclear cataracts being frequently reported alongside lamellar and powdery forms [6]. The identification of this variant in the present family, together with its segregation with the disease phenotype in the tested individuals, further supports its association with hereditary congenital cataracts. The molecular consequences of the p.(Gly91del) variant provide a plausible explanation for its association with lens opacification. Gly91 is a highly conserved residue located within the second β/γ-crystallin Greek-key motif, where it contributes to the structural organization of the crystallin domain. Deletion of this residue may interfere with the normal folding and stability of βA1- and βA3-crystallins. Previous functional studies by Reddy et al. demonstrated defective folding and reduced solubility of the Gly91del crystallin protein using in vitro protein expression and biophysical analyses [8,21]. Furthermore, Li et al. reported reduced protein expression and abnormal subcellular distribution, including increased membrane-associated aggregation of mutant CRYBA1 compared with wild-type protein [22]. More recently, Xu et al. demonstrated that the Gly91del variant increases susceptibility to protein aggregation under stress and alters βA3-crystallin oligomerization and folding behavior [23]. Together, these experimental findings support a mechanism in which altered crystallin folding and reduced solubility promote abnormal protein interactions and aggregation, potentially increasing light scattering and contributing to lens opacification. In the present study, visualization of the AlphaFold-predicted wild-type CRYBA1 structure using PyMOL provided structural context for the conserved Gly91 residue (Figure 3D), while Figure 5 schematically illustrates the proposed disease mechanism. However, protein aggregation, solubility, and stability were not directly measured in the present family, and the proposed molecular mechanism is therefore based on previously published functional evidence rather than experimental validation in patient-derived samples.

The persistent pupillary membrane observed in one of the proband’s eyes raises the question of whether the *CRYBA1* variant also affects anterior segment development. The pupillary membrane is the anterior part of the fetal vascular network that surrounds the developing lens, the tunica vasculosa lentis, and it normally regresses between approximately the sixth and eighth months of gestation [24,25]. Pupillary membrane remnants are seen in about 95% of neonates and persist in about 20% of adults [26], so a sporadic finding unrelated to the variant is plausible, although familial persistent pupillary membranes have also been described [27]. To our knowledge, persistent pupillary membrane and other forms of anterior segment dysgenesis have not been described in previously reported p.(Gly91del) families, in which the phenotype has consisted predominantly of nuclear, lamellar, or pulverulent cataracts, with nystagmus and strabismus in some affected individuals [6,8,22,28]. However, most of these reports focused on lens morphology and may not have documented subtle anterior segment findings. A biologic link is also conceivable. Beyond the lens, βA3/A1-crystallin is expressed in retinal astrocytes, and the Nuc1 rat, which carries a spontaneous *Cryba1* mutation, shows abnormal astrocyte ensheathment and failed regression of the hyaloid vasculature, resembling human persistent fetal vasculature [29,30]. In addition, experimental removal of the lens in the developing chick eye disrupts the differentiation of neural crest-derived anterior segment tissues, including the corneal endothelium and iris stroma, indicating that the lens itself provides signals that organize anterior segment development [31]. Whether structural or signaling abnormalities of a mutant lens contribute to incomplete regression of the pupillary membrane, or to subtle angle dysgenesis that could influence glaucoma risk, remains speculative. Careful documentation of the anterior segment and of fetal vascular remnants, including gonioscopy or UBM where feasible, in future *CRYBA1* families would help address this question.

Glaucoma remains a major long-term concern following pediatric cataract surgery, particularly among children undergoing intervention at a younger age [9]. It may develop during the early postoperative period or several years later, emphasizing the importance of prolonged surveillance. Longitudinal evidence from the Infant Aphakia Treatment Study demonstrated that the cumulative risk of glaucoma increased from 9% at one year to 17% at five years and 22% at ten years following unilateral cataract surgery during infancy [23,32]. Although open-angle glaucoma is commonly reported, other mechanisms, including pupillary block, have also been described [2]. The underlying pathophysiology is not fully understood, but altered development or function of the trabecular meshwork following early intraocular surgery has been proposed as a contributing factor [9]. Early recognition and management of elevated intraocular pressure are important for preventing progressive optic nerve damage [2]. Although the *CRYBA1* p.(Gly91del) variant is associated with congenital cataract formation, a direct causal relationship between this specific variant and glaucoma has not been established [8]. In the present family, the proband’s mother developed advanced glaucoma following bilateral cataract surgery during infancy, consistent with glaucoma following cataract surgery, a recognized long-term complication of pediatric cataract extraction [32]. The development of glaucoma in this context may involve alterations in anterior segment development and aqueous humor outflow following early intraocular surgery, although the underlying mechanisms remain incompletely understood. In contrast, the proband, who carries the same variant, has maintained normal intraocular pressure and has shown no evidence of glaucomatous optic nerve damage during approximately 3.5 years of postoperative follow-up (Table 2). These observations suggest that the mother’s glaucoma may be related to her history of cataract surgery during infancy rather than a demonstrated direct effect of the *CRYBA1* variant. However, the potential contribution of other genetic or developmental factors cannot be excluded, emphasizing the importance of lifelong glaucoma surveillance following congenital cataract surgery [32].

The timing of congenital cataract surgery is an important factor influencing the subsequent risk of glaucoma. The risk of glaucoma after congenital cataract surgery is highest when surgery is performed in the first weeks of life. In a cohort from Great Ormond Street Hospital, the 5-year risk of glaucoma in at least one eye after bilateral lensectomy in the first month of life was 50%, compared with 14.9% after later surgery [33], and an individual patient data meta-analysis found a twofold higher hazard of glaucoma after surgery at 4 weeks of age or younger [29]. The need for additional intraocular procedures further increases this risk [34]. The mother underwent cataract surgery at 1 to 2 months of age approximately four decades ago, before this association was widely appreciated, and her glaucoma was diagnosed shortly afterward; the glaucoma in this family may therefore reflect, at least in part, surgery very early in life. For dense bilateral cataracts, surgery is now often deferred until about 4 weeks of age when possible, while aiming to operate before about 10 weeks of age, after which the likelihood of a poor visual outcome rises [33,35]. The proband’s first surgery was performed at 8 weeks of age, with the second eye operated on one week later.

Surveillance for glaucoma after infantile cataract surgery is more challenging than in adults, and the methods must be adapted to the child’s age and cooperation. Goldmann applanation tonometry, the reference standard, requires topical anesthesia and a child who can sit at the slit lamp, fixate, and hold still, which many children can do by the preteen years. In infants, rebound tonometry (iCare) can usually be performed in the office, with a lid speculum if needed and without topical anesthesia. Children of early school age, such as a 6- or 7-year-old, fall in between. In a randomized comparison in healthy children aged 6 to 8 years, Goldmann tonometry could not be completed in 16% to 26% of children, compared with 4% to 6% with rebound tonometry, and the rebound examination took about half as long [36]. At this age, rebound tonometry or a handheld applanation device such as the Tono-Pen or Perkins tonometer is often more practical than Goldmann tonometry, although iCare readings tend to be somewhat higher than Goldmann values and should be interpreted in the clinical context [36,37]. At any age, crying, lid squeezing, or pressure on the globe while the lids are held open can falsely raise the reading, so measurements are most reliable when the child is calm. When reliable office measurements cannot be obtained, examination under anesthesia may be required, and the anesthetic regimen matters, because IOP measured under sevoflurane is substantially lower than under ketamine [38]. Interpretation is further complicated by the thicker central cornea of aphakic eyes, which can lead to overestimation of IOP [39]. In the present case, IOP at the most recent visit was measured by rebound tonometry. Because the method will likely change as the child grows, the tonometer used should be recorded at each visit so that serial measurements remain comparable.

Structural assessment of the optic nerve is similarly limited. Tabletop OCT requires steady fixation, and pediatric OCT studies have generally been limited to children of preschool age or older [40]; pediatric normative data are limited, and the high plus refraction of aphakic eyes alters scan magnification. Handheld spectral domain OCT can image the optic nerve in infants at the bedside without sedation [41], but it is not widely available. Serial optic disc photography with handheld or widefield fundus cameras, in the office or during examination under anesthesia, provides an objective record of the cup-to-disc ratio for comparison over time. Because no single measurement is sufficient, the Childhood Glaucoma Research Network requires at least two of the following findings to diagnose glaucoma: elevated IOP, optic disc changes such as progressive cupping or a cup-to-disc asymmetry of 0.2 or more, corneal enlargement or Haab striae, a myopic shift with axial elongation beyond normal growth, and reproducible visual field defects once the child is old enough to perform perimetry [42]. Serial refraction, axial length, and corneal diameter measurements are therefore useful surrogate markers in young aphakic children, and eyes that develop glaucoma after infantile cataract surgery tend to have longer axial lengths [32].

Another relevant clinical finding was intermittent esotropia, a recognized ocular alignment abnormality in children treated for congenital cataracts [3]. Strabismus may result from disrupted visual input and impaired binocular visual development, even following successful cataract extraction and optical correction [2]. Although more frequently observed in unilateral cataracts, it also occurs in children with bilateral disease [9]. Esotropia has been reported more frequently than exotropia in this population [33]. These observations highlight the importance of monitoring ocular alignment and visual development throughout childhood, with appropriate management of amblyopia or strabismus when clinically indicated [2].

An important strength of this report is the integration of clinical observations, family history, molecular diagnosis, segregation analysis, and computational structural evaluation. Nevertheless, several limitations should be acknowledged. Detailed ophthalmologic phenotyping and genetic testing were available for only a limited number of relatives, restricting comprehensive genotype–phenotype correlations within the extended family. Furthermore, the relatively short duration of postoperative follow-up in the proband limits assessment of late-onset complications, particularly glaucoma. The structural findings are also based on computational predictions and were not experimentally validated using patient-derived samples. Additional clinical evaluation of affected relatives and continued longitudinal follow-up may help clarify phenotypic variability, visual outcomes, and postoperative risks associated with this familial variant.

Overall, this case reinforces the clinical utility of molecular testing in hereditary congenital cataracts by establishing a genetic diagnosis, supporting autosomal dominant genetic counseling, and enabling targeted testing of at-risk relatives. The findings also emphasize the importance of individualized surgical management, sustained optical rehabilitation, and lifelong ophthalmologic surveillance to optimize visual outcomes and detect delayed postoperative complications.

## 4. Methodology

### 4.1. Ethics Approval and Informed Consent

This study was conducted in accordance with the principles of the Declaration of Helsinki and was approved by the Institutional Review Board of the University of Maryland, Baltimore, MD, USA (IRB: HP-00064793). Written informed consent for participation and genetic testing was obtained from the proband’s parent or legal guardian. Written consent was also obtained for publication of the clinical photographs, pedigree, ophthalmologic imaging, and de-identified genetic information presented in this study.

### 4.2. Clinical and Ophthalmologic Evaluation

The proband and his affected mother underwent comprehensive ophthalmologic evaluation at the University of Maryland Medical Center. Clinical information was obtained through review of available medical records and interviews with participating family members. A pedigree was constructed based on the reported history of congenital cataracts and related ocular findings.

The proband’s evaluation included assessment of the anterior segment, cataract laterality and severity, ocular alignment, intraocular pressure, optic nerve appearance, fundus findings on dilated examination, axial length, anterior segment and angle structure on ultrasound biomicroscopy, best-corrected visual acuity, and postoperative visual rehabilitation. External photographs were obtained before and after cataract extraction. Postoperative follow-up included monitoring of refractive correction, ocular alignment, intraocular pressure, and optic nerve appearance.

The affected mother underwent ophthalmologic examination and OCT (Heidelberg Engineering GmbH, Heidelberg, Germany) of the optic nerve and peripapillary RNFL. Sectoral and global RNFL thickness measurements were compared with the instrument’s age-matched normative database. Peripheral blood samples were collected from all participants, and genomic DNA was extracted following the standard inorganic extraction protocol [43].

### 4.3. Exome Sequencing and Variant Prioritization

Exome sequencing was performed on the proband using the Agilent SureSelect Human All Exon V6 capture kit (Agilent Technologies, Santa Clara, CA, USA), followed by sequencing on an Illumina HiSeq platform (Illumina, Inc., San Diego, CA, USA). Sequencing produced a mean coverage depth exceeding 80×, ensuring high-confidence variant detection. Raw FASTQ data underwent quality assessment using FastQC (Babraham Bioinformatics, Babraham Institute, Cambridge, UK) [44]. Reads were aligned to the hg19 (GRCh37; GCF_000001405.13) human reference genome using BWA-MEM [45]. PCR duplicates were removed using Picard Tools (Broad Institute, Cambridge, MA, USA), and variant calling was performed with GATK HaplotypeCaller (Broad Institute, Cambridge, MA, USA) [21]. Sequencing data was filtered and prioritized using the criteria described previously [46,47,48,49].

### 4.4. Sanger Sequencing and Segregation Analysis

Sanger sequencing was performed to validate the *CRYBA1* c.272_274del variant and evaluate its segregation among available participating family members. Primers flanking the genomic region containing the deletion were designed using Primer3 (https://primer3.ut.ee/, accessed on 10 October 2026) and assessed for specificity using NCBI Primer-BLAST (https://www.ncbi.nlm.nih.gov/tools/primer-blast/index.cgi?GROUP_TARGET=on, accessed on 10 October 2026) [50,51]. The primer sequences were: Forward: 5′-[AGCTCTACTGGGATTGGCTT]-3′, Reverse: 5′-[TGCCACTTGTAAGACCCTTAGT]-3′. Polymerase chain reaction amplification was performed under standard conditions using genomic DNA from the proband and available relatives. Amplified products were purified and sequenced in both directions using dye-terminator chemistry. Sequence chromatograms were aligned to the *CRYBA1* reference sequence and manually examined using DNASTAR Navigator v18 (DNASTAR, Inc., Madison, WI, USA) [52]. The variant was confirmed to be heterozygous in the proband and his affected mother.

### 4.5. Conservation and Computational Analyses

The evolutionary conservation of Gly91 was evaluated by aligning orthologous CRYBA1 protein sequences from multiple vertebrate species using the Clustal Omega online tool (EMBL-European Bioinformatics Institute, Hinxton, UK; https://www.ebi.ac.uk/jdispatcher/msa/clustalo, accessed on 10 October 2026) [53]. Conservation at the variant position was assessed using GERP++ [54], phyloP 100-way vertebrate, and phyloP 470-way mammalian scores [13]. The potential functional effect of the c.272_274del variant was evaluated using CADD and MutationTaster [12,55]. Population frequency was evaluated using gnomAD [56]. Computational predictions were considered supporting evidence and were not interpreted independently as proof of pathogenicity.

Three-dimensional (3D) structural analysis of CRYBA1 was performed using a predicted protein structure obtained from AlphaFold [57]. The structure was analyzed and visualized using PyMOL v3.1 (Schrödinger, LLC, New York, NY, USA) [58]. The conserved Gly91 residue was identified and mapped within the second β/γ-crystallin Greek-key motif. Its local structural environment and spatial relationships with neighboring amino acid residues were examined to assess the potential structural consequences of the p.(Gly91del) variant. Interatomic distances between selected neighboring residues were measured using PyMOL and expressed in Ångströms (Å). The analysis focused on local structural features and did not include quantitative calculations of folding free energy or protein stability. Schematic illustrations were created and assembled using Adobe Illustrator 2026 (Adobe Inc., San Jose, CA, USA).

### 4.6. Variant Interpretation

The *CRYBA1* c.272_274del, p.(Gly91del) variant was evaluated using the American College of Medical Genetics and Genomics and Association for Molecular Pathology guidelines, together with current ClinGen recommendations [59]. Evidence considered included population frequency, predicted protein consequences, conservation in the literature, segregation among participating family members, previous reports in individuals with congenital cataracts, and available functional evidence.

## 5. Conclusions

This case highlights the importance of integrating molecular diagnosis with timely surgical management and long-term ophthalmologic surveillance in hereditary congenital cataracts. Identification of the heterozygous *CRYBA1* c.272_274del, p.(Gly91del) variant established the molecular diagnosis in the proband and supported autosomal dominant inheritance within the family, providing clinically relevant information for genetic counseling and evaluation of at-risk relatives. Early cataract extraction followed by appropriate optical rehabilitation is important to minimize the risk of deprivation amblyopia and support visual development. Although the identified *CRYBA1* variant is associated with autosomal dominant congenital cataracts, a direct causal relationship between this variant and glaucoma has not been established. Importantly, the advanced glaucoma observed in the proband’s mother following cataract surgery during infancy underscores the need for lifelong monitoring for glaucoma and other postoperative complications in individuals treated for congenital cataracts.

## Figures and Tables

**Table 1:** Sectoral RNFL thickness analysis in the right eye of proband’s mother.

Sector	Measured Thickness	Normative Thickness	Interpretation
Temporal (T)	35 µm	75 µm	Outside normal limits (red zone)
Temporal Superior (TS)	64 µm	130 µm	Outside normal limits (red zone)
Nasal Superior (NS)	37 µm	102 µm	Outside normal limits (red zone)
Nasal (N)	34 µm	72 µm	Outside normal limits (red zone)
Nasal Inferior (NI)	66 µm	100 µm	Borderline (yellow zone)
Temporal Inferior (TI)	37 µm	143 µm	Outside normal limits (red zone)
Global (G)	43 µm	97 µm	Outside normal limits (red zone)

RNFL thickness values from a single Spectralis OCT peripapillary scan of the right eye, compared with the reference mean of the instrument’s age-adjusted European Descent (2009) normative database. The temporal, temporal superior, nasal superior, nasal, and temporal inferior sectors and the global value were outside normal limits (red zone, <1st percentile), and the nasal inferior sector was borderline (yellow zone, 1st–5th percentile), consistent with advanced glaucomatous optic neuropathy.

**Table 2:** Comparison of genetic findings, clinical manifestations, and postoperative outcomes in the proband and his affected mother.

Clinical Characteristic	Proband	Mother
*CRYBA1* variant	Heterozygous p.(Gly91del)	
Inheritance	Maternally inherited	Familial, consistent with autosomal dominant inheritance
Congenital cataracts	Bilateral	Bilateral
Age at cataract surgery	8 weeks (left eye); 9 weeks (right eye)	During infancy (around age 1–2 months)
Surgical procedure	Lens aspiration, primary posterior capsulotomy, and anterior vitrectomy; pupillary membrane excision (right eye); no primary IOL implantation	Details not available
Postoperative lens status	Aphakic	Aphakic
Glaucoma	Not detected during approximately 3.5 years of follow-up	Advanced glaucoma
Intraocular pressure	Right: 13 mmHg; Left: 14 mmHg	20 mmHg in both eyes
Optic nerve findings	No evidence of glaucomatous damage	Advanced glaucomatous optic neuropathy
RNFL thickness	Not available	Global RNFL: 43 µm (right eye)
Visual outcome	Binocular BCVA: 20/20	Advanced glaucoma-related visual impairment; BCVA 20/125 to 20/320 in the right eye and NLP in the left
Additional ocular findings	Mild esotropia	End-stage glaucoma in left eye
Glaucoma management	Continued surveillance	Multiple glaucoma drainage implants and ongoing ophthalmologic management

BCVA: best-corrected visual acuity; IOL: intraocular lens; RNFL: retinal nerve fiber layer; NLP: no light perception.

**Table 3:** In silico pathogenicity prediction analysis of the *CRYBA1* variant.

Parameter	Variant Information
Family	Eye-83
Genomic variant (GRCh37)	NC_000017.11:g.29252120_29252122del
cDNA variant	NM_005208.5:c.272_274del
Protein variant	NP_005199.2:p.(Gly91del)
Variant type	In-frame deletion
Zygosity	Heterozygous
Inheritance	Autosomal dominant; maternally inherited
gnomAD allele frequency	Not reported in the evaluated dataset
ClinVar classification	Pathogenic (Variation ID: 425127)
ACMG/AMP evidence reported by Franklin	PP1_VeryStrong, PS2, PM2, PM4, PS3_Supporting
CADD PHRED	40
GERP++	5.880
MutationTaster	0.8100 (predicted disease-causing)
phyloP 100-way vertebrate	9.54
phyloP 470-way mammalian	8.669

Ref. [12] CADD ≥20: score places mutation in top 1% of most deleterious possible substitutions; GERP++: Positive score refers to substitution deficits, suggesting evolutionary constraint (−12.3 least conserved to 6.17 most conserved). ACMG/AMP evidence criteria and their reported strengths were obtained from Franklin by QIAGEN (accessed 27 October 2026) [7]. PS2 refers to a previously reported de novo occurrence, whereas the variant was maternally inherited in family Eye-83. The reported pathogenic classification is additionally supported by ClinVar (Variation ID: 425127). Ref. [13]; phyloP 100 way Vertebrate: Conservation score using multiple alignments and a phylogenetic tree of 100 vertebrates (−20.0 least conserved to 10.003 most conserved); phyloP 470 way Mammalian: Conservation score using multiple alignments of 470 mammalian genomes including humans (−20 least conserved to 11.936 most conserved).

## Data Availability

The original contributions presented in this study are included in the report. Additional patient-level data are not publicly available because of privacy and consent restrictions. Further inquiries can be directed to the corresponding author, subject to institutional approval.

## Supplementary Materials

The following supporting information can be downloaded at: https://www.mdpi.com/article/10.3390/ijms27219978/s1.
