# Supplementary material for: Congenital Bilateral Cataracts Associated with CRYBA1: A Familial Case Report Highlighting Genetic Diagnosis, Surgical Management, and Lifelong Glaucoma Surveillance
Source: Int J Mol Sci. 2026 Nov 7;27(21):9978. doi: 10.3390/ijms27219978 (PMC13764600; doi:10.3390/ijms27219978)
Supplement: Supplementary file 1 [file ijms-27-09978-s001.zip › ijms-4674837-supplementary.pdf]

Congenital Bilateral Cataracts Associated with CRYBA1: A Familial Case Report Highlighting Genetic Diagnosis, Surgical Management, and Lifelong Glaucoma Surveillance

Mariya R. Ahmed [1], Tehmeena Akhter [2,3], Firoz Kabir [2,4], Christina Kilby [1], Andrew Nguyen [5], Osamah J. Saeedi [5] and Janet Leath Alexander [1,5,*]

[1] School of Medicine, University of Maryland, Baltimore, MD 21201, USA; mahmed@som.umaryland.edu (M.R.A.)

[2] Department of Otorhinolaryngology-Head & Neck Surgery, School of Medicine, University of Maryland, Baltimore, MD 21201, USA; takhter@som.umaryland.edu (T.A.)

[3] Centre of Excellence in Molecular Biology, University of the Punjab, Lahore 53700, Pakistan

[4] Department of Biochemistry and Biotechnology, University of Science and Technology Chittagong 4202, Chittagong, Bangladesh

[5] Department of Ophthalmology and Visual Sciences, School of Medicine, University of Maryland,
Baltimore, MD 21201, USA

* Correspondence: jalexander@som.umaryland.edu

Supplementary file:

Contains S1 and references.

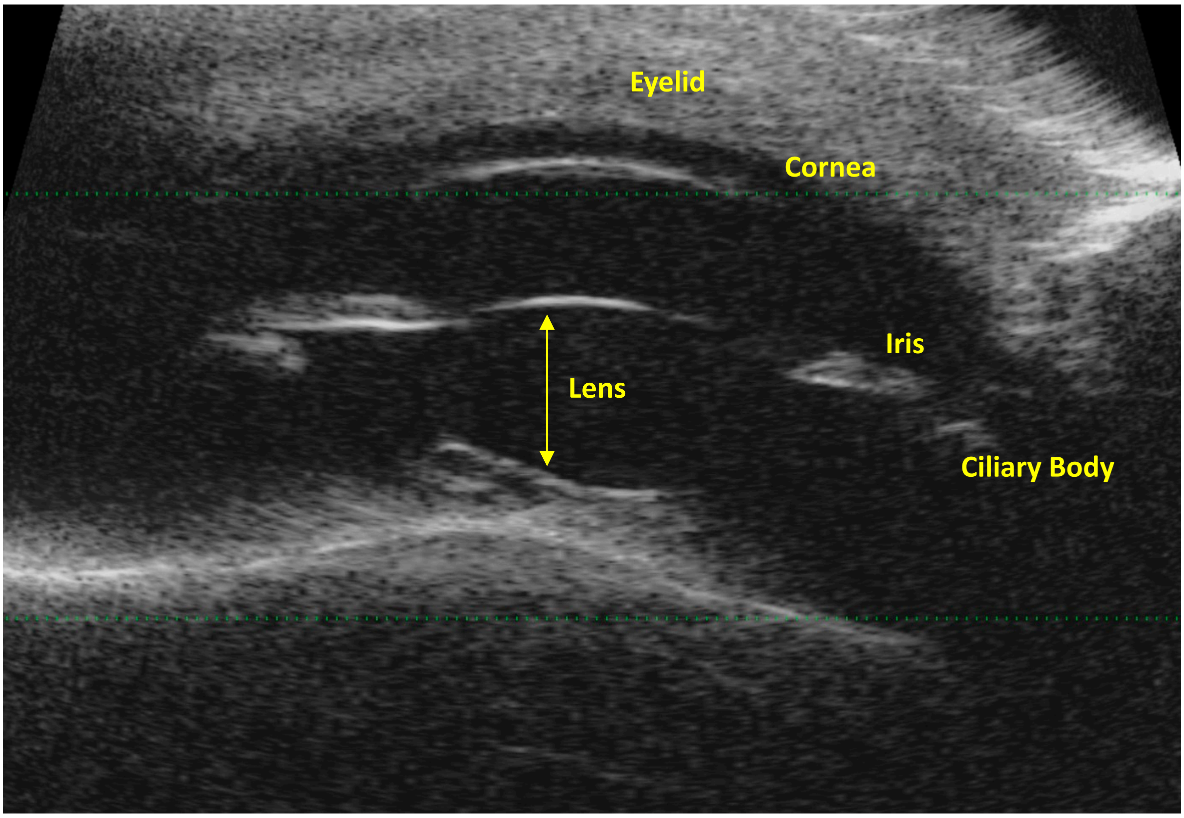

Supplement Figure S1. Ultrasound Biomicroscopy (UBM) of the left eye anterior segment, axial view, through a closed eyelid. The lens shows moderately decreased thickness, a finding frequently found in congenital cataracts [1]. The anterior chamber shows decreased depth, a finding associated with glaucoma risk following congenital cataract surgery [2].

References

1. Wei, L., et al., *Lens Thickness in Infants and Children with Cataracts.* Ophthalmology Science, 2025. **5**(1): p. 100588.
2. Wong, C., et al., *Predicting 10 Year Glaucoma Using Anterior Segment Biometry for Infants with Unilateral Congenital Cataract.* American Journal of Ophthalmology, 2026. **283**: p. 207-213.
